# Supplementary material for: Resolving the full spectrum of human genome variation using Linked-Reads
Source: Genome Res. 2019 Apr;29(4):635–45. doi: 10.1101/gr.234443.118 (PMC6442396; doi:10.1101/gr.234443.118)
Supplement: Supplemental Material [file supp_gr.234443.118_Supplemental_longranger-2.2.zip › longranger-2.2.tar.bz2!/lr2.2-public/lib/stan/cmdstan/stan/doc/api/html/structcopy__square__cholesky__dimension__if__necessary_1_1result.html]

Stan: copy\_square\_cholesky\_dimension\_if\_necessary::result< T1 > Struct Template Reference


|  |  |
| --- | --- |
|  | Stan  2.5.0  probability, sampling & optimization |


- Main Page
- Related Pages
- Namespaces
- Classes
- Files

- Class List
- Class Index
- Class Hierarchy
- Class Members

All Classes Namespaces Files Functions Variables Typedefs Enumerations Enumerator Friends Macros Pages

- copy\_square\_cholesky\_dimension\_if\_necessary
- result

Public Types |
List of all members

copy\_square\_cholesky\_dimension\_if\_necessary::result< T1 > Struct Template Reference

`#include <var_decls_grammar_def.hpp>`

|  |  |
| --- | --- |
| Public Types | |
| typedef void | type |
|  | |

## Detailed Description

### template<typename T1> struct copy\_square\_cholesky\_dimension\_if\_necessary::result< T1 >

Definition at line 510 of file var\_decls\_grammar\_def.hpp.

## Member Typedef Documentation

template<typename T1 >

typedef void copy\_square\_cholesky\_dimension\_if\_necessary::result< T1 >::type

Definition at line 510 of file var\_decls\_grammar\_def.hpp.

---

The documentation for this struct was generated from the following file:

- src/stan/gm/grammars/var\_decls\_grammar\_def.hpp


---

[ Stan Home Page ]

*© 2011–2014,
Stan Development Team.*
